# Enzymatic Synthesis and Characterization of Hydrophilic Sugar Based Polyesters and Their Modification with Stearic Acid

**DOI:** 10.3390/polym8030080

**Published:** 2016-03-16

**Authors:** Muhammad Humayun Bilal, Marko Prehm, Andrew Efraim Njau, Muhammad Haris Samiullah, Annette Meister, Jörg Kressler

**Affiliations:** Department of Chemistry, Martin Luther University Halle-Wittenberg, D-06099 Halle (Saale), Germany; muhammad.bilal@chemie.uni-halle.de (M.H.B.); Marko.prehm@chemie.uni-halle.de (M.P.); andrew.njau@student.uni-halle.de (A.E.N.); muhammad.samiullah@chemie.uni-halle.de (M.H.S.); annette.meister@chemie.uni-halle.de (A.M.)

**Keywords:** enzymatic synthesis, biodegradable polyesters, sugars, graft copolymers, stearic acid, crystallization, nanoparticles

## Abstract

Biodegradable and hydrophilic functional polyesters were synthesized enzymatically using xylitol or d-sorbitol together with divinyl adipate and lipase B from *Candida antartica* (CAL-B). The resulting polyesters had pendant OH-groups from their sugar units which were esterified to different degrees with stearic acid chloride. The structure and the degrees of polymerization of the resulting graft copolymers based on poly(xylitol adipate) and poly(d-sorbitol adipate) were characterized by ^1^H NMR spectroscopy and SEC. DSC, WAXS and SAXS measurements indicated that a phase separation between polymer backbone and stearoyl side chains occurred in the graft copolymers, and, additionally, the side chains were able to crystallize which resulted in the formation of a lamellar morphology. Additionally, nanoparticles of the graft copolymers in an aqueous environment were studied by DLS and negative stain TEM.

## 1. Introduction

Aliphatic functional polyesters are an important class of materials for pharmaceutical and biological applications because of their biocompatibility and biodegradability [1]. The enzymatic synthesis of functional polyesters is an attractive alternative to classical polycondensation or ring opening polymerization, which require an extensive protection and deprotection chemistry for the introduction of functional groups to the polyester backbone [2]. The most frequently used enzyme for this purpose is lipase B from *Candida antarctica* (CAL-B) which is commercially available immobilized on acrylic resins [3]. Since CAL-B supports the esterification of primary OH-groups rather than secondary OH-groups at reasonably low temperatures, it is frequently employed to prepare linear polyesters from polyols as glycerol or sugars [4,5,6,7,8], as second reactants usually divinyl esters [6,9], or dimethyl esters [7,8,10] are employed. A typical polyester with a pendant OH-group in every monomer unit is poly(glycerol adipate) (PGA) obtained by reacting glycerol with divinyl adipate [6]. We have extensively studied PGA grafted with several fatty acids. Self-assembled nanoparticles obtained from these amphiphilic polymers have been studied for drug delivery applications [11]. Different shapes of nanoparticles could be produced in water by the interfacial deposition method depending on the degree of substitution and on the fatty acid used. Important for the structure formation of the nanoparticles is the phase separation between polymer backbone and side chains [11,12]. Saturated fatty acids with relatively long acyl chains (>C12) tend to crystallize after phase separation whereas unsaturated fatty acids as, e.g., oleic acid lead to the formation of two disordered nanophases. The PGA backbone obtained by enzymatic polymerization is hydrophilic but not water soluble and has usually *M*_n_-values below 10.000 g·mol^−1^. It should be mentioned that also higher molar masses can be achieved at higher polymerization temperatures but then the selectivity between primary and secondary OH-groups is partially lost [13]. Grafting poly(ethylene oxide) (PEO) chains onto the PGA backbone results in water soluble polymers [14]. When a block copolymer of PEO and poly(ε-caprolactone) is grafted to the polymer backbone, worm-like micelles can be produced [15]. The PGA backbone can also be converted into an ATRP macroinitiator, which leads to a plethora of possible polymer architecture [16,17].

**Scheme 1:**
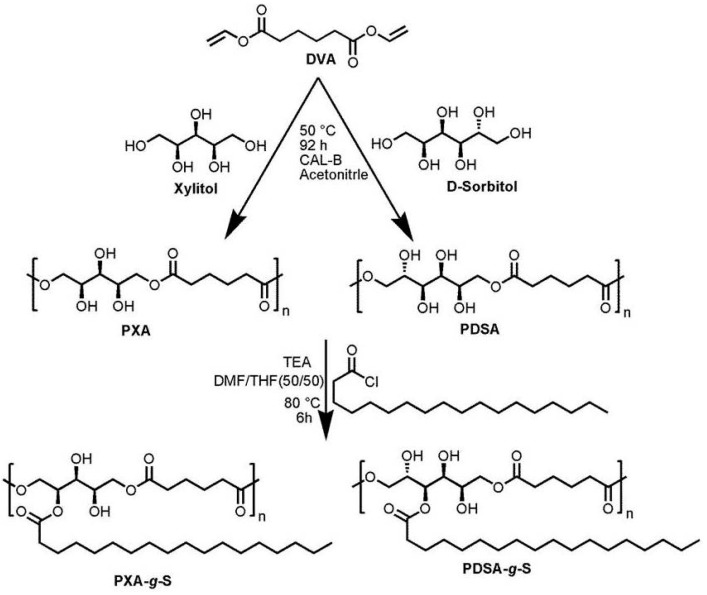
Synthetic route to stearoyl grafted poly(xylitol adipate) (PXA-*g*-S) and poly(d-sorbitol adipate) (PDSA-*g*-S).

Here, we report on the enzymatic syntheses of poly(d-sorbitol adipate) (PDSA) and poly(xylitol adipate) (PXA). Both hydrophilic polyester backbones were modified by grafting with stearic acid chloride which yields amphiphilic polymers (PDSA-*g*-S and PXA-*g*-S). All polymers are characterized by SEC and ^1^H NMR spectroscopy. The bulk morphology of the polymers is studied by DSC, WAXS and SAXS. Furthermore, nanoparticles are prepared from some of the polymers and characterized by DLS and TEM. Scheme 1 describes the syntheses of the polymers under investigation.

## 2. Materials and Methods

### 2.1. Materials

Lipase N435 derived from *Candida antarctica* (CAL-B) immobilized on acrylic resin (Sigma-Aldrich, St. Louis, MO, USA) was dried over P_2_O_5_ for 24 h prior to use. *N,N*-dimethylformamide (anhydrous, 99.8%), acetonitrile (anhydrous, 99.8%), tetrahydrofuran (anhydrous, 99.9%), xylitol (99%) and d-sorbitol (98%), stearoyl chloride (90%) and triethylamine were purchased from Sigma-Aldrich. Divinyl adipate (96%) was purchased from TCI GmbH (Eschborn, Germany). All chemicals were used without further purification. All other chemicals used in various stages like, diethyl ether, n-hexane, methanol and dichloromethane were purchased from Carl Roth (Karlsruhe, Germany).

### 2.2. Syntheses

#### 2.2.1. Synthesis of Poly(xylitol adipate)

Analogous to previously published methods, poly(xylitol adipate) (PXA) can be synthesized in a single step [4,18]. Xylitol and divinyl adipate were used in a molar stoichiometric ratio of 1:1 in the presence of lipase N435. A weighed amount of xylitol (10 g, 65.8 mmol) and divinyl adipate (13 g, 65.8 mmol) were charged into the reaction flask. Afterwards, 40 mL acetonitrile was added. The solution was stirred for 30 min at 50 °C to equilibrate the temperature. The reaction was started by adding CAL-B (2.3 g, 10% *w*/*w* of total mass of monomer). The reaction was quenched by addition of an excess of DMF after 92 h and then the enzyme was removed by filtration. The synthesis process is described in Scheme 1. The filtrate was collected and the crude product was precipitated twice in diethyl ether. ^1^H NMR spectroscopy was used to confirm the purity of the sample. ^1^H NMR (400 MHz, DMSO-d_6_) δ (ppm): 4.86–4.76 (m, 2H), 4.68 (dd, J = 18.6, 7.6 Hz, 1H), 4.11–3.92 (m, 4H), 3.73 (s, 2H), 3.38 (s, 1H), 2.29 (s, 4H), 1.52 (s, 4H).

#### 2.2.2. Synthesis of Poly(d-sorbitol adipate)

Poly(d-sorbitol adipate) (PDSA) was synthesized using the same procedure as described for poly(xylitol adipate) but the crude product was dialyzed to remove oligomers using water as dialysis medium and a membrane with a cut-off molar mass of 1000 g∙mol^−1^. ^1^H NMR spectroscopy was used to confirm the purity of the sample. ^1^H NMR (400 MHz, DMSO-d_6_) δ (ppm): 4.97–4.61 (m, 2H), 4.55–4.30 (m, 2H), 4.28–3.82 (m, 3H), 3.70 (d, J = 49.1 Hz, 2H), 3.58–3.33 (m, 2H), 2.37–2.17 (m, 4H), 1.64–1.42 (m, 4H).

#### 2.2.3. Synthesis of Stearoyl Grafted Poly(Xylitol Adipate) and Poly(d-sorbitol adipate)

Poly(xylitol adipate) and poly(d-sorbitol adipate) with a molar mass *M*_n_ of 5000 g·mol^−1^ and 3500 g·mol^−1^, respectively, were used for further modification with stearoyl chains using a standard procedure. Under continuous nitrogen flow, PXA or PDSA was dissolved in DMF/THF (50/50) was injected into a three neck 100 mL round bottom flask, equipped with condenser and magnetic stirrer. Triethylamine was added as an acid scavenger. Stearoyl chloride was dissolved in THF at room temperature and added dropwise to the reaction mixture. The temperature was raised to 80 °C, and the reaction was stopped after 6 h. The solvent was removed in a rotary evaporator. To remove the salt of trimethylamine and unreacted trimethylamine, the crude product was dissolved in DCM and extracted four times with brine. The organic phase was collected and dried with magnesium sulfate. The crude product was precipited in n-hexane for products less than 40% grafted chains and in methanol for products with more than 40% grafting, to remove unreacted fatty acid. A slight brownish solid product was obtained. PXA-*g*-S: ^1^H NMR (500 MHz, CDCl_3_) δ (ppm): 5.47–4.99 (m, 3H), 4.54–3.7 (m, 4H), 2.35 (d, J = 42.4 Hz, 10H), 1.67 (s, 10H), 1.26 (s, 84H), 0.89 (t, J = 6.9 Hz, 9H). PDSA-*g*-S: ^1^H NMR (400 MHz, CDCl_3_) δ (ppm): 5.65–4.76 (m, 4H), 3.5–4.5 (m, 4H), 2.35 (m, 12H), 1.64 (s, 12H), 1.43–1.15 (m, 112H), 0.87 (t, J = 6.8 Hz, 12H).

### 2.3. Preparation of Nanoparticles

Nanoparticles were prepared using the interfacial deposition method describe elsewhere [6] with slight modification. In this approach 150 µL of 15 mg·mL^−1^ polymer solution in THF was added slowly to 1.7 mL hot water (above the melting temperature of the polymer) with constant vortexing for 5 min. Turbid dispersions were obtained which were further homogenized using silentCrusher S (Heidolph Instruments GmbH, Schwabach, Germany) for 3 min. 300 µL cold water was then added for immediate solidification of the particles with vortexing. The organic solvent and small amounts of water were evaporated in a rotary evaporator. A small amount of water was again added to make 0.1% *w*/*v* dispersion.

### 2.4. NMR Spectroscopy

^1^H NMR spectra were measured with a Varian Gemini 400 spectrometer at 400 MHz and 25 °C. Approximately 30 mg of polymer were dissolved in 0.8 mL of CDCl_3_ or DMSO-d_6_ as solvents, purchased from Armar Chemicals (Döttingen, Switzerland). The NMR spectra were interpreted using Mest Rec v.4.9.9.6 (Mestrelab Research, Santiago de composstella, Spain). The assignment of the peaks was made using Chem Office 2004-Chemdraw ULTRA 8 software from Cambridge, UK.

### 2.5. Size Exclusion Chromatography (SEC)

SEC measurements were performed on a ViscotekGPCmax VE 2002 using HHRH Guard-17360 and a GMHHR-N-18055 columns and refractive index detector (VE 3580 RI detector, Viscotek, GmbH, Waghäusen, Germany). THF was used as eluent. For all samples, the concentration was 3 mg·mL^−1^ and the flow rate was 1 mL·min^−1^. For PXA and PDSA, the measurements were done with DMF containing 0.01 M LiBr as mobile phase in a thermostated column oven kept at 80 °C. The calibration standards for measurements in THF and DMF were polystyrene and poly(methyl methacrylate), respectively.

### 2.6. Differential Scanning Calorimetry (DSC)

Thermal properties of the polymers were measured using a differential scanning calorimeter (DSC, Mettler Toledo DSC823e module, Mettler-Toledo GmbH,Greifensee, Switzerland). All samples were scanned within the temperature range of −50 to 80 °C with a heating rate of 10 °C·min^−1^. 8 mg to 12 mg of sample material were enclosed in an aluminum pan and placed in the DSC sampler at room temperature. All measurements were performed under the constant flow of nitrogen (10 mL·min^−1^).

### 2.7. Dynamic Light Scattering (DLS)

All samples were measured in Plastibrand PMMA disposable cuvettes (Brand GmbH, Wertheim, Germany) with the ALV-NIBS/HPPS spectrometer (ALV-Laser Vertriebsgesellschaft M.B.H., Langen, Germany). As light source, an He–Ne laser with a power of 3 mW and a wavelength of λ = 632.8 nm was used. The scattering intensity was recorded by a photomultiplier at an angle of 173°, and the temporal intensity fluctuations were recorded with a Multiple Tau digital correlator ALV transfer 5000/E (Vertriebsgesellschaft M.B.H., Langen, Germany). ). The analysis was performed with the ALV Correlator Software 3.0 through the “regularized fit” mode.

### 2.8. Negative-Staining TEM

Samples for negative-staining TEM were prepared by spreading 3 µL of the dispersion onto a Cu grid coated with a Formvar^®^ film (Plano, Wetzlar, Germany). The excess liquid was blotted off after 30 s. Afterwards, the grid was placed on a droplet of 1 wt % aqueous uranyl acetate solution and drained off after 1 min. The dried specimens were observed with a Zeiss EM 900 transmission electron microscope (Carl Zeiss Microscopy GmbH, Oberkochen, Germany). Micrographs were taken with a SSCCD SM-1K-120 camera (TRS, Moorenweis, Germany).

### 2.9. X-Ray Diffraction (XRD)

X-ray diffraction patterns were recorded with a 2D detector (Vantec 500, Bruker, AXS, Madison, WI, USA) using Ni filtered and pinhole collimated Cu K_α_ radiation. The exposure time was 15 min and the sample to detector distance was 9.85 and 27.7 cm for wide and small angle scattering experiments, respectively.). The samples were held in glass capillaries (ø 1 mm, Hilgenberg, GmbH, Malsfeld, Germany).

## 3. Results and Discussion

### 3.1. Polymerization and Grafting

The enzymatic syntheses of PXA and PDSA are carried out by polycondensation reaction of xylitol and d-sorbitol, respectively, with divinyl adipate. Since the enzyme reacts preferably with primary hydroxyl groups at reasonably low temperatures, the secondary hydroxyl groups remain unreacted [18]. As a result, linear PXA and PDSA with pendant OH-groups are obtained. PDSA is a water soluble polymer whereas PXA forms a fine suspension in water. Dynamic light scattering of a 10 g·L^−1^ suspension of PXA is given in Figure S1 (Supplementary Materials).

**Figure 1:**
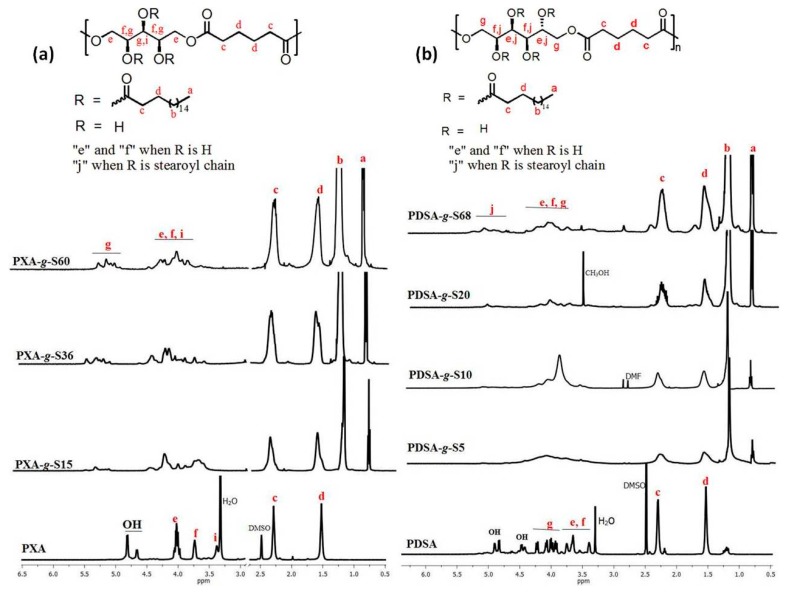
^1^H NMR spectra of (**a**) grafted and non-grafted poly(xylitol adipate) and (**b**) grafted and non-grafted poly(d-sorbitol adipate), recorded at 25 °C using CDCl_3_ and DMSO-d_6_ as solvent for grafted and non-grafted polymers, respectively.

In the second step of synthesis, the functional polyesters are grafted with stearoyl chains resulting in different degrees of grafting. ^1^H NMR spectra of grafted and non-grafted PXA and PDSA are shown in Figure 1. The relative increase in intensity of the peaks belonging to the stearoyl side chains indicates the degree of grafting. The quantitative determination of degree of grafting is calculated by taking into account the integral values of peaks “a” and “c” using Equation (1) for PXA-*g*-Sx and Equation (2) for PDSA-*g*-Sx.

(1) $$\text{\% degree of grafting}=\;\frac{0.44\;\times \;a}{c-0.67a}\times 100,$$

(2) $$\text{\% degree of grafting}=\;\frac{0.33\;\times \;a}{c-0.67a}\times 100.$$

**Figure 2:**
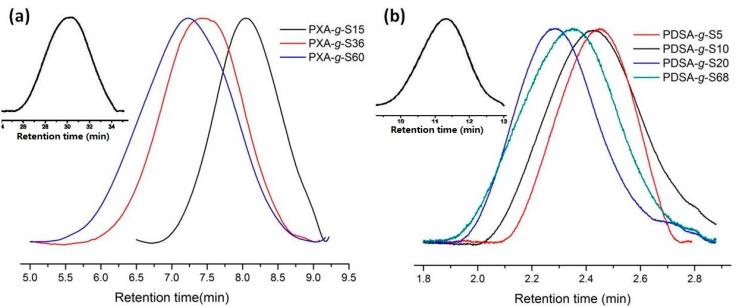
Size exclusion chromatography (SEC) traces of (**a**) stearoyl grafed poly(xylitol adipate) in THF; (**b**) SEC traces of stearoyl grafted poly(d-sorbitol adipate) in THF. The insets show the SEC traces of the polymer backbones PXA and PDSA, respectively, in DMF.

Using Equations (1) and (2), the degrees of grafting calculated for PXA was 15%, 36% and 60%, whereas for PDSA, the respective values are 5%, 10%, 20% and 68%. The grafting is further verified by size exclusion chromatography (see Figure 2), where a shift of the peak maximum to lower retention times with increasing degrees of grafting occurs.

Due to the insolubility of polymer backbones PXA and PDSA in THF, the measurements are carried out in DMF as eluent while for all other grafted polymers THF is employed. The number average molar masses and the polydispersity index (PDI) defined as *M*_w_/*M*_n_ together with thermal properties of all polymers are given in Table 1.

**Figure 3:**
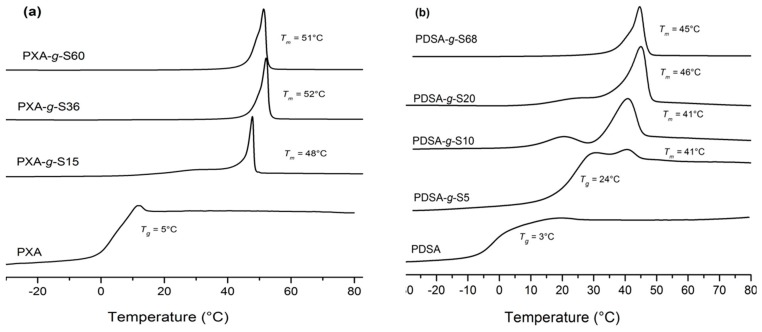
DSC traces of (**a**) grafted and non-grafted PXA and (**b**) grafted and non-grafted PDSA. The measurements are carried out with a heating rate of 10 K·min_−1_.

Thermal properties of grafted and non-grafted PXA and PDSA, respectively, are determined by differential scanning calorimetry. PXA and PDSA are amorphous polymers with a glass transition temperature *T*_g_ of 5 and 3 °C, respectively. However, all graft copolymers are semicrystalline indicated by the melting endotherms. All details on the phase transitions are summarized in Table 1. The melting temperature *T*_m_ is determined from the maximum of the endothermal peak during the second heating scan. DSC traces of graft copolymers and polymer backbones are shown in Figure 3.

As already mentioned, the grafting of saturated fatty acids to this type of polyesters induces crystallinity caused by side-chain crystallization. The melting temperature is about 50 °C in case of PXA-*g*-Sx, whereas for PDSA-*g*-Sx, the melting peak occurs between 41 and 45 °C. For very low degrees of grafting, both a glass transition temperature and a melting endotherm can be observed in the DSC traces.

**Figure 4:**
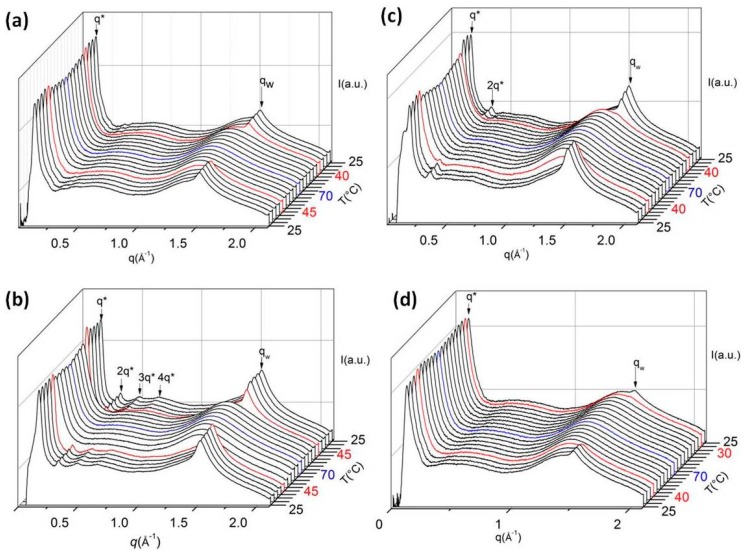
Combined SAXS and WAXS traces during heating and cooling cycles (5 K step from 25 °C to 70 °C and back to 25 °C) of (**a**) PXA-*g*-S15; (**b**) PXA-*g*-S36; (**c**) PDSA-*g*-S68; and (**d**) PDSA-*g*-S10.

Combined SAXS and WAXS investigations are carried out for polymers in bulk. These comb-like polymers show nanophase separation that arises due to phase separation of the side chains from the immiscible polymer backbone [19,20]. Ordering of these nanophases strongly depends on the lipophilic volume fraction of the side chains [12]. For polymers having a relatively low degree of grafting, a low volume fraction of the side chains is obtained, for instance PXA-*g*-S15 (Figure 4a) has a volume fraction of the side chains of 0.37. For this sample, a peak in the small angle regime at *q* = 0.1136 Å^−1^ (the corresponding d-spacing is 55.3 Å) during heating scan is observed. In the wide angle region only one single peak is visible (*q*_w_ = 1.5222 Å^−1^) superimposed by diffuse scattering. Assuming a hexagonal ordering of the side chains, this peak corresponds to the (110) direction of the hexagonal lattice with orthorhombic setting, having a lattice parameter *a* = 4.77 Å (hexagonal rotator phase R_II_) [12,21]. At the melting temperature of 48 °C, the peak in the wide angle region disappears whereas an amorphous halo remains at lower *q*-values and the peak in the small angle region becomes broader.

For polymers with relatively high degrees of grafting, a more ordered nanophase separation is observed, for instance, PXA-*g*-S36 (the volume fraction of hydrophobic side chain is 0.58). Combined SAXS and WAXS heating and cooling scans of PXA-*g*-S*36* are shown in Figure 4b. At low temperature, one prominent peak in the small angle region (q*) and several higher order peaks are visible, indicating a lamellar structure caused by nanophase separation of the polymer backbone and the side chains (*d* = 38.1 Å) followed by crystallization of side chain [12]. In the wide angle region, only one single peak appears (*q*_w_). The lattice parameter *a* = 4.78 Å is nearly identical with that of PXA-*g*-S15. At the melting temperature of 52 °C, the peak in the small angle region becomes broader and the higher order peaks disappear. This behavior indicates that the lamellar structure is significantly stabilized by the side chain crystallization. After cooling below the crystallization temperature of 45 °C, the higher order peaks appear again.

The sample PDSA-*g*-S10 (Figure 4c) with a volume fraction of 0.30 of side chains shows a similar scattering pattern as PXA-*g*-S15 with a slightly larger d-spacing of 57.1 Å. The sample PDSA-*g*-S68 (Figure 4d) with higher side chain content (volume fraction of 0.71) shows in the small angle regime again the typical scattering pattern of a lamellar phase with a d-spacing of 35.5 Å. The complete data of all SAXS and WAXS measurements are given in Tables S1–S8 in the Supplementary Materials. It is interesting to note that the lattice parameters of the hexagonal rotator phase remains the same, independent of the degree of grafting.

### 3.2. Nanoparticles

#### 3.2.1. Dynamic Light Scattering

**Figure 5:**
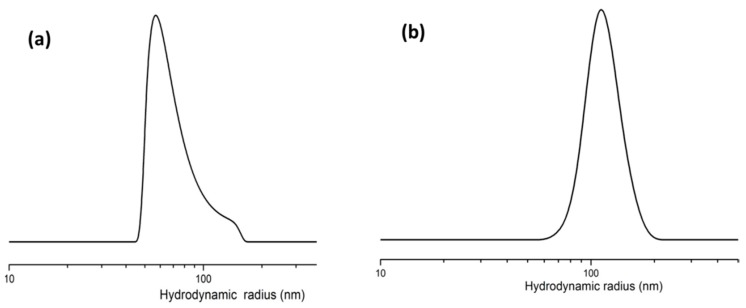
(**a**) Hydrodynamic radius distribution of PXA-*g*-S15 nanoparticles in water with a concentration of 1 g∙L^−1^ at 25 °C; (**b**) hydrodynamic radius distribution of PDSA-*g*-S10 nanoparticles (1g∙L^−1^ in water at 25 °C).

Dynamic light scattering is employed to determine the size of nanoparticles formed by the graft copolymers. The polymer dispersion in water at a concentration of 1 g∙L^−1^ at 25 °C is used. The average hydrodynamic radius of PXA-*g*-S15 is found to be 69 nm whereas for PDSA-*g*-S10 it is 105 nm. The reason behind the difference in particle sizes of both samples could be the overall hydrophilicity of the polymer chains. Since PDSA-*g*-S10 is more hydrophilic than PXA-*g*-S15, it shows a comparatively higher hydrodynamic radius in water. The particle size distributions of the respective polymer dispersions are shown in Figure 5. Since the size and shape of the nanoparticles are important factors for *in vivo* drug release [11,22,23], the control of these parameters is important. The size of the nanoparticles can be tailored by choosing appropriate solvents and temperatures for the interfacial deposition method. Any solvent that is miscible with water and that can dissolve the polymer as well will be appropriate for this method [24]. A solvent with poor water miscibility results in a decrease in interfacial tension during the preparation process and results in an increase in particle size [25].

#### 3.2.2. Negative-Staining Transmission Electron Microscopy

**Figure 6:**
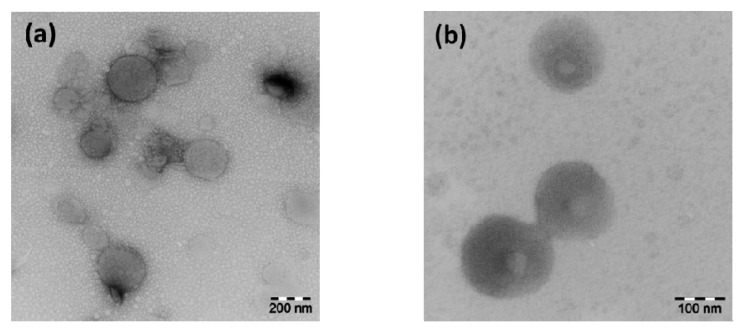
Negative-staining electron micrographs, (**a**) 0.1% dispersion of PXA-*g*-S15; (**b**) 0.1% dispersion of PDSA-*g*-S10.

To study the morphology of the nanoparticles, negative-staining TEM on dried nanoparticles was carried out using uranyl acetate as the staining agent. The negative stained TEM images are shown in Figure 6.

Figure 6 shows spherical morphologies of the nanoparticles, which is a typical behavior of particles formed upon surface energy minimization. It can be seen that PXA-*g*-S15 forms homogeneous nanoparticles whereas for PDSA-*g*-S10 some kind of structuring in the nanoparticles is observed. Since the geometry, surface properties and internal structure of nanoparticles strongly influence the drug incorporation [23], studies are in progress to investigate these characteristics in our graft copolymer systems.

## 4. Conclusions

In conclusion, a simple approach for synthesizing sugar based functional polyesters has been used to produce hydrophilic polymers, which were grafted with stearoyl chains to obtain amphiphilic grafted polyesters. Unlike the initial amorphous polyester backbone, the grafted polyesters are semicrystalline in nature. These amphiphilic comb-like polymers show a nanophase separation leading to lamellar morphologies which are more pronounced in the case of relatively high degree of grafting, such as for PXA-*g*-S36 and higher. However, for small degree of grafting this lamellar nanophase separation is less ordered, which results in only one peak in the SAXS traces. A diffraction peak at higher scattering angles (WAXS) in all polymer samples corresponds to the (110) reflection of the hexagonal phase that exists until the melt temperature of the polymers is reached. Furthermore, these polymers are able to form well-arranged nanoparticles. It would be interesting, in the future, to explore the morphology and properties as, e.g., the hydrolytic stability of the nanoparticles formed from the these polymers and to develop their applications in drug delivery system. Due to the biodegradability of these nanoparticles, they might be an alternative to many Pluronic^®^ based systems.

## Figures and Tables

**Table 1:** Volume % of side chains, *M*_n_, polydispersity index (PDI), glass transition temperature (*T*_g_) melting temperature (*T*_m_) and melting enthalpy (*ΔH*_m_) of polymers.

Polymer	Volume fraction of stearoyl chains ^5^	*M*_n_ (g·mol^−1^)	PDI	*T*_m_ (°C) ^4^	*T*_g_ (°C) ^4^	*ΔH*_m_ (J·g^−1^) ^4^
**PXA**	-	5,000 ^1^	2.4 ^1^	-	5	
**PXA-*g*-S15**	0.37	7,300 ^2^	2.3 ^3^	48	20	60
**PXA-*g*-S36**	0.58	10,600 ^2^	2.6 ^3^	52	-	100
**PXA-*g*-S60**	0.69	14,600 ^2^	1.8 ^3^	51	-	85
**PDSA**	-	3,500 ^1^	1.9 ^1^	-	3	-
**PDSA-*g*-S5**	0.15	4,300 ^2^	1.4 ^3^	41	24	1.15
**PDSA-*g*-S10**	0.30	4,800 ^2^	1.9 ^3^	41	-	36
**PDSA-*g*-S20**	0.43	5,800 ^2^	1.5 ^3^	46	-	136
**PDSA-*g*-S68**	0.71	12,200 ^2^	1.7 ^3^	45	-	73

^1^ *M*_n_ and Polydispersity index (PDI) are obtained from SEC using DMF as eluent; ^2^ *M*_n_ is calculated on the basis of % degree of grafting from ^1^H NMR spectra; ^3^ PDI is obtained from SEC using THF as eluent. ^4^ Obtained from DSC using a heating rate of 10 K·min^−1^; ^5^ Volume fraction of the stearoyl chains were calculated by software, Materials Studio 4.1.

## Supplementary Materials

Supplementary materials can be found at www.mdpi.com/2073-4360/8/3/80/s1.

## Abbreviations

The following abbreviations are used in this manuscript:

PEO	Poly(ethylene oxide)
PGA	Poly(glycerol adipate)
PXA	Poly(xylitol adipate)
PDSA	Poly(d-sorbitol adipate)
PXA-*g*-Sx	Poly(xylitol adipate)-*graft*-stearoyl chain (x represents % degree of grafting)
PDSA-*g*-Sx	Poly(d-sorbitol adipate)-*graft*-stearoyl chain (x represents % degree of grafting)
SAXS	Small angle X-ray scattering
WAXS	Wide angle X-ray scattering
SEC	Size exclusion chromatography
DSC	Differential scanning calorimetry
DLS	Dynamic light scattering
