# Supplementary material for: Enzymatic Synthesis and Characterization of Hydrophilic Sugar Based Polyesters and Their Modification with Stearic Acid
Source: Polymers (Basel). 2016 Mar 16;8(3):80. doi: 10.3390/polym8030080 (PMC6432536; doi:10.3390/polym8030080)
Supplement: Supplementary file 1 [file polymers-08-00080-s001.pdf]

Supplementary Materials: Enzymatic Synthesis and Characterization of Hydrophilic Sugar Based Polyesters and Their Modification with Stearic Acid

Muhammad Humayun Bilal, Marko Prehm, Andrew Efraim Njau, Muhammad Haris Samiullah, Annette Meister and Jörg Kressler

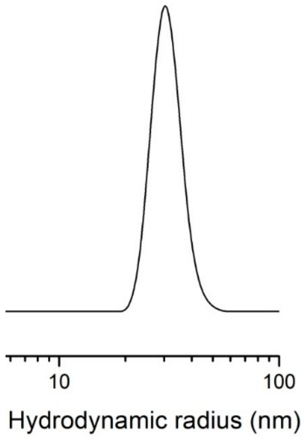

Figure S1. Hydrodynamic radius distribution of PXA at concentration 10 g·L^{-1} at temperature 25 °C.

Table S1. WAXS data of PXA-*g*-S15.

***T* (°C)**	***q* (A^{-1})**	***d* (A)**	
**30 °C heating**	1.3663	4.599	diffuse
	1.5222	4.128	q_w
**70 °C**	1.3817	4.547	diffuse
**30 °C cooling**	1.4138	4.444	diffuse
	1.5204	4.133	q_w

Table S2. SAXS data of PXA-*g*-S15.

***T* (°C)**	***q* (A^{-1})**	***d* (Å)**	
30 °C heating	0.1136	55.310	q^*
70 °C	0.1284	48.934	Broad Peak
30 °C cooling	0.1230	51.083	q^*

q^* is the wave vector at the first maximum.

Table S3. WAXS data of PXA-*g*-S36.

***T* (°C)**	***q* (A^{-1})**	***d* (Å)**	
**30 °C heating**	1.5187	4.137	q_w
**70 °C**	1.3661	4.599	diffuse
**30 °C cooling**	1.5174	4.141	q_w

Table S4. SAXS data of PXA-*g*-S36.

T (°C)	*q* (Å^{-1})	*d* (Å)	
**30 °C heating**	0.1648	38.126	q^*
	0.3286	19.121	$2q^*$
	0.4903	12.815	$3q^*$
**70 °C**	0.1676	37.489	Broad Peak
**30 °C cooling**	0.1649	38.103	q^*
	0.3292	19.086	$2q^*$
	0.4896	12.833	$3q^*$

q^* is the wave vector at the first maximum.

Table S5. WAXS data of PDSA-*g*-S10.

T (°C)	*q* (Å^{-1})	*d* (Å)	
**30 °C heating**	1.4299	4.394	diffuse
	1.5199	4.134	q_w
**70°C**	1.3788	4.557	diffuse
**30 °C cooling**	1.3557	4.635	diffuse
	1.5015	4.185	q_w

Table S6. SAXS data of PDSA-*g*-S10.

T (°C)	*q* (Å^{-1})	*d* (Å)	
30 °C heating	0.1100	57.120	q^*
70 °C	0.1205	52.143	Broad Peak
30 °C cooling	0.1133	55.456	q^*

q^* is the wave vector at the first maximum.

Table S7. WAXS data of PDSA-*g*-S68.

T (°C)	*q* (Å^{-1})	*d* (Å)	
**30 °C heating**	1.5150	4.147	q_w
**70 °C**	1.3640	4.606	diffuse
**30 °C cooling**	1.5123	4.155	q_w

Table S8. SAXS data of PDSA-*g*-S68.

T (°C)	*q* (Å^{-1})	*d* (Å)	
**30 °C heating**	0.1734	36.235	q^*
	0.3464	18.139	$2q^*$
**70 °C**	0.1856	33.853	Braod Peak
**30 °C cooling**	0.1768	35.538	q^*
	0.3506	17.921	$2q^*$

q^* is the wave vector at the first maximum.

© 2016 by the authors; licensee MDPI, Basel, Switzerland. This article is an open access article distributed under the terms and conditions of the Creative Commons by Attribution (CC-BY) license (http://creativecommons.org/licenses/by/4.0/).
